# Practical and Theoretical Considerations in Study Design for Detecting Gene-Gene Interactions Using MDR and GMDR Approaches

**DOI:** 10.1371/journal.pone.0016981

**Published:** 2011-02-28

**Authors:** Guo-Bo Chen, Yi Xu, Hai-Ming Xu, Ming D. Li, Jun Zhu, Xiang-Yang Lou

**Affiliations:** 1 Institute of Bioinformatics, Zhejiang University, Hangzhou, Zhejiang, People's Republic of China; 2 Section on Statistical Genetics, Department of Biostatistics, University of Alabama at Birmingham, Birmingham, Alabama, United States of America; 3 Department of Psychiatry and Neurobehavioral Sciences, University of Virginia, Charlottesville, Virginia, United States of America; Aarhus University, Denmark

## Abstract

Detection of interacting risk factors for complex traits is challenging. The choice of an appropriate method, sample size, and allocation of cases and controls are serious concerns. To provide empirical guidelines for planning such studies and data analyses, we investigated the performance of the multifactor dimensionality reduction (MDR) and generalized MDR (GMDR) methods under various experimental scenarios. We developed the mathematical expectation of accuracy and used it as an indicator parameter to perform a gene-gene interaction study. We then examined the statistical power of GMDR and MDR within the plausible range of accuracy (0.50∼0.65) reported in the literature. The GMDR with covariate adjustment had a power of>80% in a case-control design with a sample size of≥2000, with theoretical accuracy ranging from 0.56 to 0.62. However, when the accuracy was<0.56, a sample size of≥4000 was required to have sufficient power. In our simulations, the GMDR outperformed the MDR under all models with accuracy ranging from 0.56∼0.62 for a sample size of 1000–2000. However, the two methods performed similarly when the accuracy was outside this range or the sample was significantly larger. We conclude that with adjustment of a covariate, GMDR performs better than MDR and a sample size of 1000∼2000 is reasonably large for detecting gene-gene interactions in the range of effect size reported by the current literature; whereas larger sample size is required for more subtle interactions with accuracy<0.56.

## Introduction

Complex traits are controlled by multiple genetic factors working in concert and responding to the environment. Although the exact inheritance mechanisms of such traits are largely unknown, it is commonly accepted that there are interactions of numerous biological processes, which contribute, directly or indirectly, to phenotypes [1], [2]. These genetic mechanisms differ from those of conventional Mendelian traits in several ways: (1) multiple genes are involved [3], [4]; (2) the roles of the genes are defined in the context of their related genes; and (3) the magnitude of the gene effects depends on the environment to which they are exposed [5]. A major achievement in detecting epistasis for complex traits is the development of constructive induction approaches [6], including the multifactor dimensionality reduction method (MDR) [7], [8], [9], the combinatorial partitioning method (CPM) [10], and the restricted partition method (RPM) [11]. The MDR is a powerful approach to detect gene-gene (G×G) interactions and ideally discriminates between discrete clinical endpoints when using multilocus genotypes [12]. To circumvent the weaknesses of existing MDR approaches [13], we previously developed a generalized MDR (GMDR) statistical framework applicable to both dichotomous and quantitative phenotypes that allows adjustment for covariates in population-based study designs [14]. We then extended our approach to family-based designs with pedigree-based GMDR (PGMDR) [15], and other extensions of it are emerging [16], [17], [18]. So far, MDR and its extensions have identified many interacting genetic variants underlying various complex human diseases, such as Alzheimer disease [19], asthma [20], atrial fibrillation [21], autism [22], bladder cancer [23], hypertension [24], nicotine dependency [14], [15], [25], [26], prostate cancer [27], [28], schizophrenia [29], sporadic breast cancer [7], thrombotic stroke [30], and Type II diabetes [31], [32] (see Table S1 for details).

Statistical power is a key factor to consider when an investigator designs a trial. Although there is a vast literature on power analysis for single-factor approaches [33], [34], [35], [36], [37], [38], [39], [40], [41], fewer studies have explored the statistical power of MDR and its extended approaches to detect interactions. A thorough study of power for interaction detection under various theoretical assumptions is thus warranted, as statistical power depends on the specific experimental scenario defined by factors such as sample size, significance level, penetrance, population prevalence, allele frequencies, interaction orders, interaction patterns, and sampling scheme, all of which are difficult to determine exactly and can be evaluated only by simulations. To reflect the reality as much as possible for gene-gene interaction studies, we assessed statistical power through intensive simulations of hypothetical scenarios with regard to the information in the literature.

The primary purpose of this study was to examine the statistical power for detecting G×G interactions in case-control designs using GMDR and MDR approaches through simulating various scenarios with the goal of providing empirical guidelines for designing such studies. Although it is generally preferred to use the traditional parameters such as heritability and genotype-relative-risk (GRR) [42] to characterize experimental scenarios, we propose using accuracy as an indicator parameter to capture the characteristics of an ascertained population. We demonstrate that accuracy is practically estimable and Testing Accuracy (TA) converges to theoretical accuracy in a large sample. Furthermore, we establish an empirical link between TA and heritability.

## Materials and Methods

### Methods

Although the MDR and GMDR methods, as well as the underlying terminology, have been presented in the literature [7], [9], [14], we offer a brief summary here to enable readers to follow our presentation easily. In general, these methods share the same framework [9] (Figure S1). In step one, the dataset is partitioned randomly into 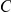 equal or nearly equal subdivisions. (We use 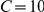 throughout this report.) One subdivision is used as the testing set and the rest as the independent training set. In step two, a subset of 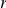 discrete genetic or environmental factors is selected from all 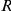 factors of interest. We have 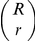 combinations exhaustively. In step three, the training set stretches into 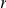-dimensional space, and each genotyped subject is allocated to a cell accordingly. The values of the score statistic can be summed in each cell. Here, the GMDR differs from the MDR in which the numbers of cases and controls are directly employed. Without adjustment for covariates, the GMDR is reduced to MDR [14]. Each non-empty cell is then labeled as either high-risk, if the average statistic value is not less than a preset threshold *T*, or low-risk otherwise. In step four, an interaction model is created by pooling high- and low-risk cells into distinct groups. Some fitness measure is then assessed. Without loss of generality, here we used accuracy (i.e., classification accuracy in step four and TA in step six), although other appropriate measure can also be used. Balanced accuracy may be a better alternative in unbalanced data sets [43]. In step five, all other possible combinations of 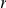 factors in the training set are examined, and the best 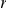-factor model with the maximum classification accuracy is recorded. In step six, the best model from step five is evaluated for TA by the testing set. There are 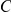 pairs of training-testing sets, so the above procedure is repeated independently 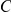 times on the sets, and the best models are ranked.

As both the MDR and the GMDR use classification accuracy to identify the best model and TA to evaluate the goodness of fit, we examine here the property of ‘accuracy’, which is defined as
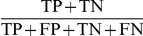
where TP is true positive having a high-risk value in the high-risk group, TN is true negative with a low-risk value in the low-risk group, FP is false positive, and FN is false negative. When other metrics are used such as sensitivity 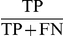, specificity 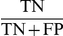, and balanced accuracy 
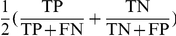
, they can be evaluated similarly. For an ascertained population, accuracy is a better characteristic parameter than heritability or GRR because even the same heritability or GRR can result in various genotype distributions with different allele frequencies, prevalences, penetrances, and ascertainment schemes. Further, accuracy is a natural measure for the contribution rate of genes of interest because we do not intend to estimate heritability and GRR parameters in the nonparametric MDR and GMDR approaches. In what follows, we use the logistical model to elucidate accuracy through constructing a conditional genotypic distribution and conditional score distributions and then to calculate the mathematical expectation of accuracy.

### Logistic model for a dichotomous trait

For a complex trait, in addition to a functional genotypic combination, environmental factors affect penetrance. We construct a general penetrance function by considering genotypic and covariate effects together. For a dichotomous phenotype, 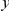, affected subjects are coded 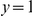 and unaffected 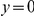. Assume the dichotomous trait 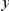 has a Bernoulli distribution with the probability 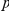 for a subject being affected; this situation can be modeled with a generalized linear model:



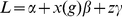
(1)where 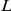 is a logit link function, 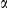 is the intercept, 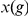 is the coding for genotype 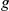, 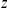 is the coding for the covariate, and 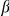 and 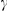 are the corresponding parameters, respectively. Given the 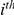 subject, the probability of being affected is:
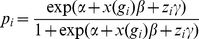
(2)


The GMDR is based on the use of the residual score of model (1), defined as:
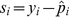
(3)


where 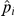 is estimated from Equation (2) where 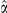 and 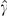 are their maximum likelihood estimates (MLE) in model (1) under the null hypothesis *H*_0_: 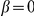.

### Conditional genotype and score distributions

To derive the theoretical accuracy, we first focus on the genotype distribution for a case-control sample. Consider the case sample by repeated application of Bayes' theorem; for genotype 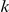, we have:
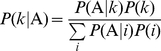
(4)


where 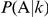 is the probability of being affected for a given genotype 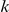, 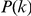 is the prior probability of genotype 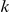 in the population from which the sample comes, and the denominator is the sum of the numerator over all genotypes. By applying Equation (4) to the control sample, for a given genotype 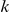, we obtain 
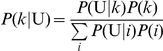
, where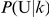 is the probability of an unaffected subject and 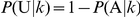. Under the null hypothesis, the penetrances are the same for all genotypes, and thus Equation (4) can be simplified to 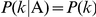 for both case and control samples. In contrast, under the alternative hypothesis, the value of 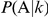 depends on genotype 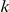. For complex traits, it is likely that covariate(s) are involved in their etiologies, and thus 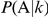 is further determined by the environmental factor, say, 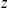, so that: 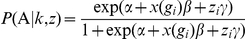, as presented in Equation (2). 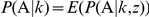, which is obtained by the integral of the expression over variable 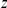 given its probability density function 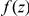:
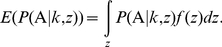
(5)


**Figure 1:**
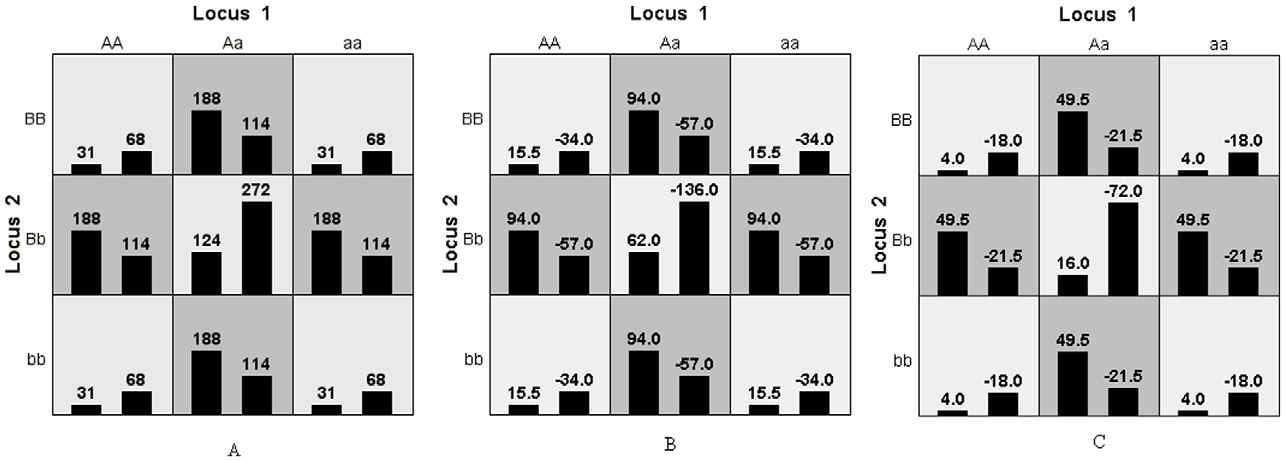
Conditional genotype and score distributions. (A): Conditional genotype distribution; (B): Conditional score distribution without covariate adjustment; and (C): Conditional score distribution with covariate adjustment. The parameters used in our simulations under the balanced case-control design are: 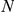 = 2000, MAF = 0.5, 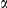 = -5.30, 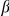 = 2.5, 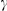 = 1, and 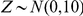.

To demonstrate the method, we offer the theoretical genotype distribution for a checkerboard model scenario, as commonly employed in this type of interaction study [14], [44], [45]. In the following sections, we consider a penetrance function containing only one covariate, but when necessary, it can easily be extended by incorporating more covariates and other effects; e.g., gene × environment factors. We assume a balanced case-control design with 2000 unrelated subjects, MAF = 0.5, 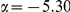, 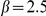, 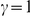, and a covariate 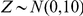. Under such assumptions, the trait is expected to have a heritability of 0.043 (according to the definition of Culverhouse et al. [44]), and there are two differential risk genotypic groups with their expected penetrances of 0.073 and 0.221 (0.005 and 0.057 if the covariate is excluded), which can be calculated from Equation (5) through numerical solution. After applying these equations, we obtain the expected genotype distribution for the case-control sample, as presented in Figure 1A (see Text S1 for details on calculating this distribution). Such an approach of generating the conditional genotype distribution is flexible and can be applied easily to other scenarios. When no covariate is considered, as assumed in the MDR approach [46], the genotype distribution becomes a simpler form.

The sums of the affected and unaffected scores in genotypic cell 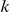 can be calculated as: 
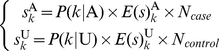
(6)respectively, where 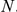 is the number of the cases or the controls and 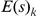 denotes the expectations of the score of an affected or an unaffected subject given genotype 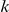. 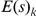 can be computed, respectively:
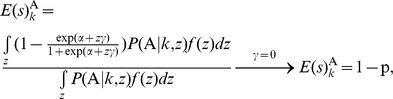



and
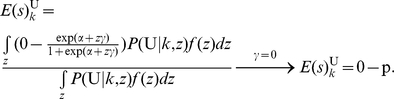



In the case without adjustment by the covariate (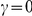), these two equations can be simplified, where p is the prevalence of the disease in the sample with its expectation 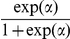. Figures 1B and 1C show the score distributions without and with covariant adjustment, respectively (see Text S2 for details on calculating the distributions illustrated in Figure 1C). Although only one covariate was adjusted in the derivation of the score distribution, such adjustment of the covariate is necessary and can be applied to cases with more than one covariate.

### Accuracy and Testing Accuracy

As defined, the TA always ranges from 0.5 to 1.0. For the GMDR method with and without covariate adjustment, the accuracies for the case shown in Figures 1B and 1C are 0.648 and 0.743, respectively. Indeed, as discussed previously [14], without adjustment of covariates, the accuracy can be estimated directly from the conditional genotype distribution. This has been confirmed by the identical values of other statistics calculated from the distribution in Figures 1A and 1B.

**Figure 2:**
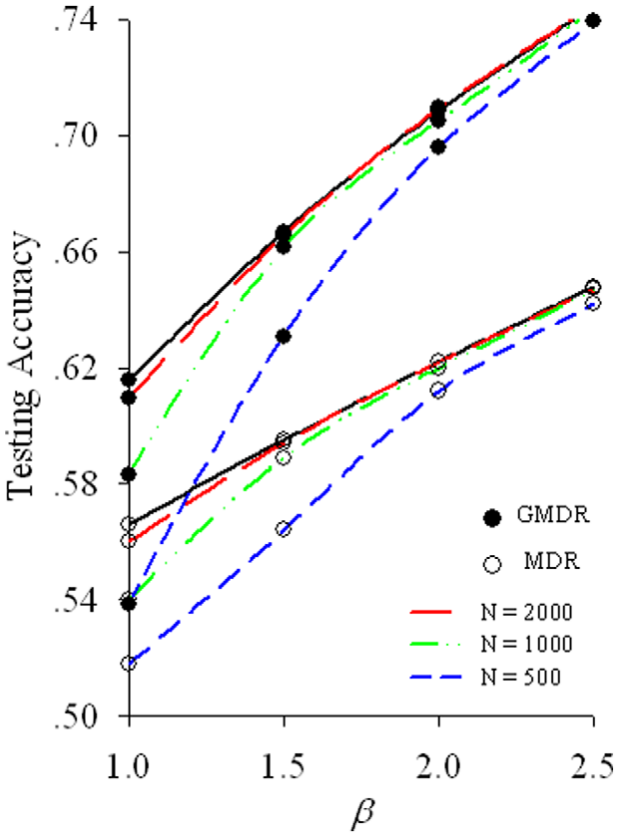
Asymptotic trends of testing accuracy with different sample sizes. The result was based on a checkerboard model whose parameters were the same as shown in Figure 1. The solid lines are the analytical accuracy and represent the upper bound of the testing accuracy. The three lines downward are the means of the testing accuracies from 200 simulations with a sample size of 2000, 1000, and 500. Because the lines for a sample size of>2000 are coincident with the analytical accuracy lines, they are not shown.

TA is commonly used in GMDR and MDR. Because it is context-dependent, its mathematical expectation is difficult to derive straightforwardly. Empirically, we show in Figure 2 that when the sample size increases to infinity under a checkerboard model, TA approaches accuracy, which is the theoretical upper bound of TA. For the cases illustrated, TA closely converges to accuracy with a sample size of 1000∼2000. The upper limit of TA can be attained when, in the testing set, each genotypic cell is recognized correctly as high or low risk after the cell has been classified correctly in the training set.

### Simulations

As approximately 85% of detected interactions involved more than one, but less than four genetic loci (Table S1), in this report, we present only the results from three interaction models on the basis of 10 diallelic loci: one digenic (i.e., two functional gene loci involved), one trigenic (i.e., there functional loci involved), and one tetragenic (i.e., four functional loci involved). For convenience of notation, loci are denoted by different letters and the two alleles at each locus by uppercase and lowercase; e.g., *A* and *a* for locus 1, *B* and *b* for locus 2, etc. For the digenic model, the checkerboard, which was commonly used in epistatic studies because of its weak marginal effects, was employed [14], [44], [45]. As elucidated previously, accuracy can serve as an indicator statistic to guide experimental design, so we relaxed the definition of the detailed genetic architecture of high-order interaction and focused on the TA a model can reach. For simplicity, we used models called the 3 uppercase letter model (3ULM), in which genotypes with 3 uppercase letters were set as high risk (e.g., AaBbCc, AABbcc, AAbbCc), and the 4 uppercase-letter model (4ULM), in which genotypes containing 4 uppercase letters were set as high risk for tetragenic interaction.

We employed a balanced experimental design with three moderate sample sizes (500, 1000, and 2000) and two large samples (4000 and 10,000) because large samples have been more prevalent in many recent reports [47], [48], [49]. To cover a broad spectrum, we set three levels (0.10, 0.25, and 0.50) of minor allele frequency (MAF) for interacting loci. Hardy-Weinberg and linkage equilibria were assumed throughout the simulations.

Our simulated populations followed the penetrance function defined in Equation (2) where 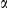 is the intercept with a value of -5.30, 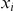 is the predictor variable coding for G×G interaction, and 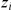 is the covariate with a normal distribution 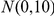. Our simulated genotypic effects were 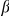 = 1.0, 1.5, 2.0, and 2.5, respectively, and 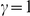. We investigated three interaction models, four levels of 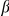, three levels of MAF, and five sample sizes. There were 180 scenarios in total for our simulation study. For each scenario, we simulated 200 replications in order to produce a precise evaluation of statistical power.

To calculate statistical power, we needed to determine the threshold for each scenario under GMDR and MDR, respectively. For GMDR, we shuffled the residual scores to generate pseudo-samples under the null hypothesis of no association with interaction, and TA was evaluated for each set of pseudo-samples. After repeating this procedure 1000 times and ranking the 1000 TAs obtained, the threshold for TA at a 5% significance level can be determined for the scenario under investigation. The power was calculated by the proportion of the true models identified in 200 simulations with a TA larger than the threshold evaluated for this scenario. The best model was identified on the maximization of average TA and cross-validation consistency (CVC) according to the principle of parsimony that the simplest model is preferred, and the simpler interaction model was chosen if the two statistics suggested different models. The permutation procedure was similar for MDR to calculate the statistical power, except for shuffling the phenotypic values instead of the residual scores obtained with adjustment of the covariate in GMDR. Such a protocol was commonly used in other reported power studies on the MDR method [45], [46].

The GMDR software was used to detect gene-gene interactions under various scenarios. The default setting of parameters was adopted in this study, and the GMDR software was also used to conduct MDR algorithm by converting the status of each individual to the corresponding score without covariate adjustment.

## Results

**Figure 3:**
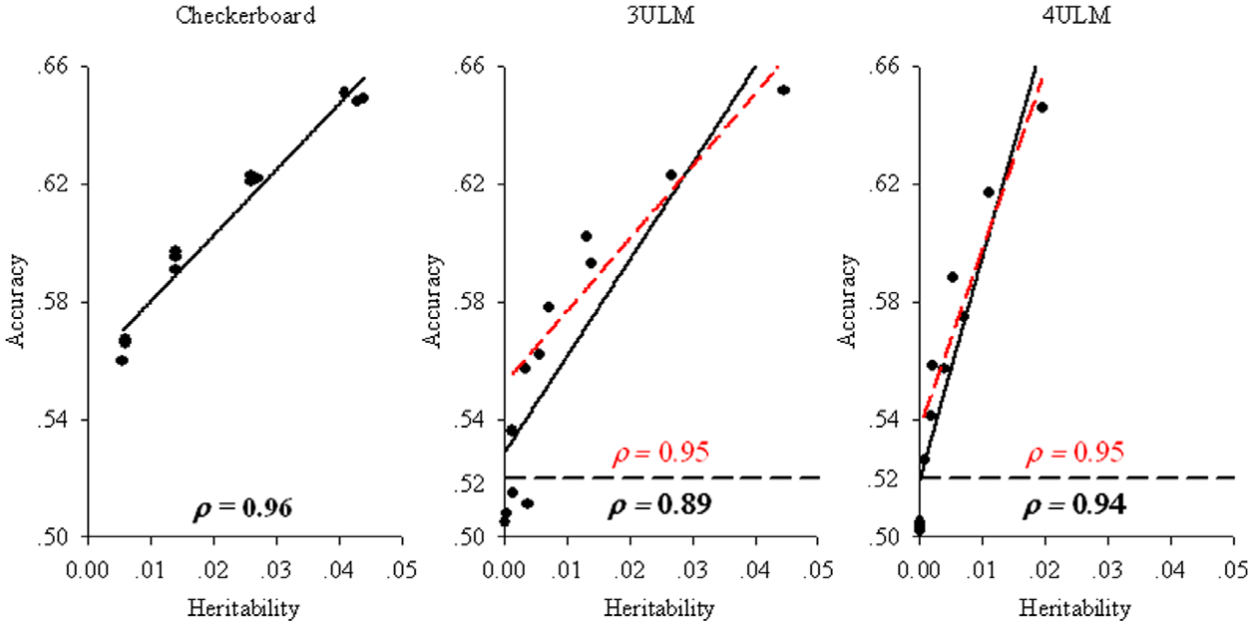
Linear correlation between accuracy and heritability. The solid line in each panel is fitted with the method of least squares, and its 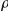 is shown in bold font. The dashed lines in 3ULM and 4ULM panels were fitted alike while excluding dots below 0.52, and their 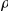 values are shown above the reference lines indicating accuracy of 0.52. For the six regression models, the *p* value for *F* test was<0.001.

For comparison of the three models, their accuracies were calculated by the aforementioned method (Table 1). The heritability under each scenario was calculated, and the relations between accuracy and heritability are plotted in Figure 3. Because each interaction underlying a complex trait often contributes only a small fraction to the overall heritability, the estimated heritability for any single interaction is<0.05. In addition, there appears to be a linear correlation between accuracy and heritability, with an 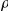 (coefficient of correlation) ranging from 0.89 to 0.98 for the three models (Figure 3). If we excluded accuracies below 0.52, where MAF = 0.1, 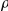 increased for both 3ULM and 4ULM, especially for the 3ULM model, with 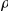 increasing from 0.89 to 0.95 (Figure 3). There were many G×G interactions detected underlying human diseases (Figure S2, and Table S1), in which mostly the strength of the interactions was measured by TA, rather than heritability. When applying the linear correlation obtained from simulations to the interactions detected by MDR and its extended methods, we predict that the corresponding heritability for most detected gene-gene interactions is between 0.01 and 0.05.

**Table 1:** Theoretical accuracies for the three simulated models^a^.

Model	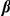	MAF		
		0.1	0.25	0.5
Checkerboard	1.0	0.560	0.567	0.566
	1.5	0.591	0.597	0.595
	2.0	0.621	0.623	0.622
	2.5	0.649	0.651	0.648
3ULM	1.0	0.505	0.536	0.562
	1.5	0.508	0.557	0.593
	2.0	0.511	0.578	0.623
	2.5	0.515	0.602	0.652
4ULM	1.0	0.502	0.526	0.558
	1.5	0.503	0.541	0.588
	2.0	0.504	0.557	0.617
	2.5	0.505	0.575	0.646

a Accuracies were calculated on the basis of the conditional genotypic distribution or of the score distribution without adjustment. For each model, three levels of MAFs and four genotype effects were employed. Hardy-Weinberg and linkage equilibria were assumed.

Generally speaking, for the three interaction models simulated, the proportion of wrong models that were significant at the 5% level was close to 0.05, as expected (data not shown). Furthermore, most wrong models contained one or more functional loci, and therefore, the wrong models could be treated as partially detected.

**Figure 4:**
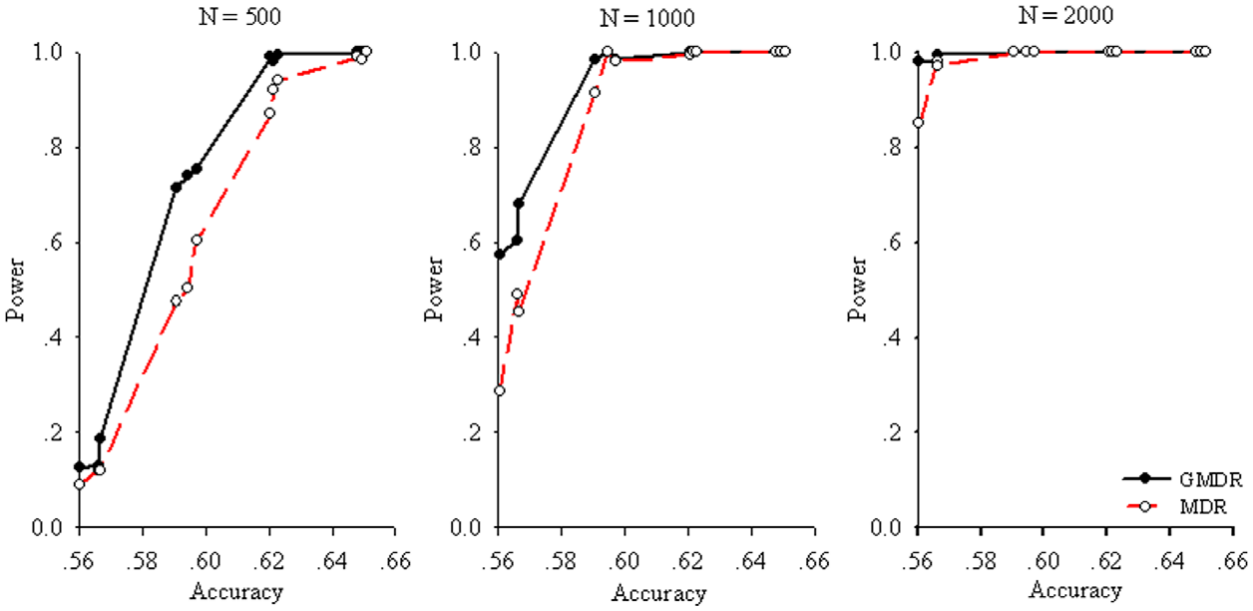
Power comparison of GMDR and MDR for sample sizes of 500, 1000, and 2000 under the checkerboard (digenic) model at alpha = 0.05. For each panel, 12 combinations, as defined in Table 1, were simulated, forming three levels of MAF (0.1, 0.25, and 0.5) and four levels of interactive effects (1.0, 1.5, 2.0, and 2.5). Simulation results from sample sizes of 4000 and 10,000 are not shown because no difference in power estimates were detected by the GMDR and MDR methods.

Figure 4 presents the powers of GMDR and MDR for the checkerboard model. As shown, the GMDR had at least 80% for a sample size of≥1000, when the theoretical accuracy is around 0.56∼0.62. This appears to be true for a sample of 500 when the accuracy is>0.60. It is clear that the GMDR outperformed MDR in most scenarios. This was attributed mainly to adjustment of the covariate in the GMDR. However, such an advantage diminished when the accuracy was>0.62, as both the GMDR and the MDR methods showed almost full power. This was also true for a larger sample (i.e., 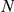
>2000; data not shown).

**Figure 5:**
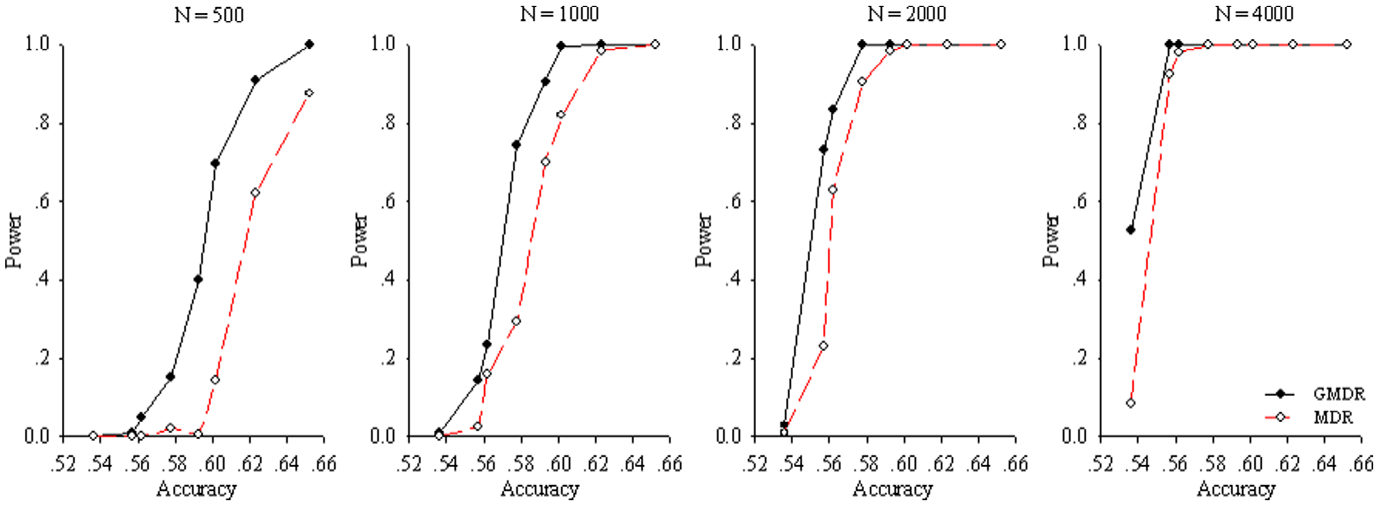
Power comparison of GMDR and MDR for sample sizes of 500, 1000, 2000, and 4000 under the 3ULM (trigenic model) at alpha = 0.05. For each panel, 12 combinations, as defined in Table 1 were simulated, as shown here, which were formed of three levels of MAFs (0.1, 0.25, and 0.5) and four levels of interaction effects (1.0, 1.5, 2.0, and 2.5).

**Figure 6:**
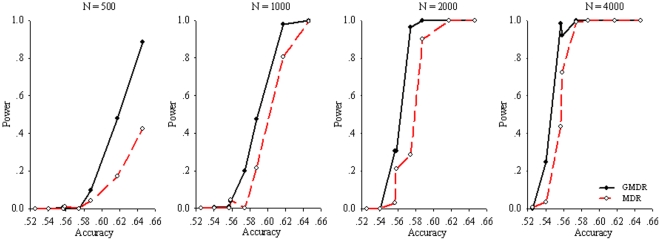
Power comparison of GMDR and MDR for sample sizes of 500, 1000, 2000, and 4000 under the 4ULM (tetragenic model) at alpha = 0.05. For each panel, 12 combinations, as defined in Table 1, were simulated, as shown here, which were formed of three levels of MAFs (0.1, 0.25, and 0.5) and four levels of interaction effects (1.0, 1.5, 2.0, and 2.5). Simulation results from the sample of 10,000 are not shown because no difference in power estimates was detected for the GMDR and MDR methods.

Figures 5 and 6 show the powers for the 3ULM and 4ULM. As shown in Table 1, because the accuracy is<0.52 when MAF = 0.1, the power results for those scenarios are less meaningful and thus will not be presented. Similar to the results in the digenic model, the GMDR outperformed MDR when the accuracy was between 0.56∼0.62, and it was more apparent for 3ULM at sample sizes of 500 and 1000. For the GMDR, in order to yield a power greater than 80% efficiently with accuracy at 0.56, a reasonable sample size should be at least 2000 for trigenic and 4000 for tetragenic models.

## Discussion

Widespread but elusive multifactor interactions usually result in a weak marginal correlation between a factor and the phenotype, posing a significant challenge in identification of the risk factors for complex diseases. Increasing effort is being expended to design powerful detection methods. Although several promising methods are available, the relevant issues of study design and data analysis for detecting interactions including sample size for a desirable power and the efficiency of statistical methods have not been well explored. Here, we compared the statistical power and the accuracy of two commonly used methods, MDR and GMDR through theoretical computation and simulation studies under a broad range of sample sizes and hypothetical parameter settings in which the real parameters would potentially fall. The results provide an empirical guideline for investigators to plan appropriate studies.

In previous power studies of MDR [43], [46], [50], heritability was commonly employed as an indicator parameter. As heritability depends not only on genotypic penetrance and disease prevalence but also on genotypic frequencies in a studied population, it is a measure both of the population and of the gene effects — in other words, heritability is a population-specific parameter even for the same phenotype. Often, if not always, the original reference and an ascertained population show differences in allele frequencies, and heritability measured from the original population is not sensitive in reflecting the property of an ascertained population and vice versa. In case-control designs, thus, heritability is an indicator parameter of less theoretical and practical value.

We believe that accuracy is a better metric to characterize the connection between sample size and power in an interaction study. First, in both GMDR and MDR, the classification accuracy and TA are computed directly from the sample. Second, classification accuracy and TA converge asymptotically to the theoretical accuracy and can offer an estimate of accuracy with a reasonably large sample. Third, accuracy is a straightforward and comprehensive measure of the strength of causality and the goodness of fit of the model, through which other factors such as gene frequencies, gene effects, heritability, and ascertainment conditions influence statistical power. To gain a better understanding of accuracy, we developed a general analytical method to compute the mathematical expectation of accuracy, previously investigated *in silico*, for a case-control sample. As an indicator statistic, accuracy worked well in our simulations. It should be noticed that balanced accuracy suggested by Velez et al [43] may be a better metric to measure the fitness when the numbers of cases and controls are unequal for the MDR method.

Furthermore, we found an empirical linear correlation between accuracy and heritability in a wide range of circumstances given different MAFs and penetrances under balanced case-control designs. This will help find a connection between the previous reports [7], [11] and the present study. For the cases simulated and under the sampling scheme investigated, accuracy ranging from 0.55∼0.65 can be converted to a heritability of 0.01∼0.05. This implies that most of the interactions in the literature, the TAs of which fell in this range (Figure S2), have a heritability of 0.01∼0.05 with a sample size of 1000 to 2000. This correlation provides an interpretation of genetic meaning for interactions detected by GMDR and MDR, and probably is applicable to interactions detected by other nonparametric statistics, such as balanced testing accuracy [43].

In this study, we evaluated the statistical power of GMDR and MDR using accuracy as an indicator to determine the sample sizes required to provide sufficient testing power in a case-control design. The GMDR with covariate adjustment could have a power of>80% for an unrelated case-control design with a sample size≥2000, whereas the theoretical accuracy is around 0.56∼0.62; when the accuracy is<0.56 (heritability close to 0.01), a sample size of at least 4000 would be required to provide sufficient power. Generally speaking, when the sample size was 1000∼2000, GMDR appeared to outperform MDR for all simulated models within the accuracy range, from 0.56 to 0.62, which was close to the densely distributed region of TA in the published data (Figure S2). As the sample size became larger, their difference became less obvious. Large samples will become more common in the near future, although most studies have a sample size of<2000. The benefit of large samples in improving statistical power and detecting interactions of much smaller effect sizes may be validated in the future. As argued recently, however, tiny effects are increasingly discovered in genome-wide association studies with the help of enlarged samples, but whether tiny effects are of great interest remains unclear [51], [52]. Balancing the sample size and the significance of the interaction detected deserves consideration, such as in Figure S3, yet more data are needed to confirm that the strength of interactions decreases in tandem with the sample size.

Although the above results were obtained entirely on the basis of a case-control design, it can be introduced into the discordant sib pair design because of their similarity in population structure. For quantitative traits, as the process of gaining the mathematical expectation of accuracy should be derived differently, it requires an additional endeavor to reach similar conclusions and consequently mandates further work. It seems difficult, although probable, that in the future, interaction studies will move to the genome-wide scale [53], and consequently the choice of genotyping chips [41] and imputation approaches [54] should be considered.

The GMDR software which was initially released in 2007 [14] and now is available at http://www.ssg.uab.edu/gmdr.

## Supporting Information

Figure S1 Six steps involved in data reduction algorithm.
(TIF)

Figure S2 A distribution of testing accuracy from the recently reported literature on gene-gene interactions detected by the MDR or GMDR approaches, with a mean of 0.606, SD of 0.047, and range of 0.50 to 0.70 (Shapiro-Wilk test: *p* = 0.8033). The width of each bin is 0.02. A detailed list of these studies yielding the values used in this study is provided in Table S1.
(TIF)

Figure S3 Scatter plot of reported gene-gene interactions with respect to their testing accuracy and sample sizes. The vertical lines partition the literature into four intervals with respect to their sample sizes: (0, 500), (500, 1000), (1000, 2000), and (2000, 4000). The location of each black circle is determined by the means of testing accuracy and sample size over the open spots within each interval flanked by two neighboring vertical lines. Because of the limited information available, the open circle for the sample size of≥4000 is not shown.
(TIF)

Table S1 Testing accuracy of human diseases detected with GMDR/MDR methods in the recent literature.
(DOC)

Text S1 Conditional genotype distribution of the checkerboard model.
(DOC)

Text S2 The expectation of the residual score for a subject.
(DOC)
