# Supplementary figures and images for: Practical and Theoretical Considerations in Study Design for Detecting Gene-Gene Interactions Using MDR and GMDR Approaches
Source: PLoS One. 2011 Feb 28;6(2):e16981. doi: 10.1371/journal.pone.0016981 (PMC3046176; doi:10.1371/journal.pone.0016981)

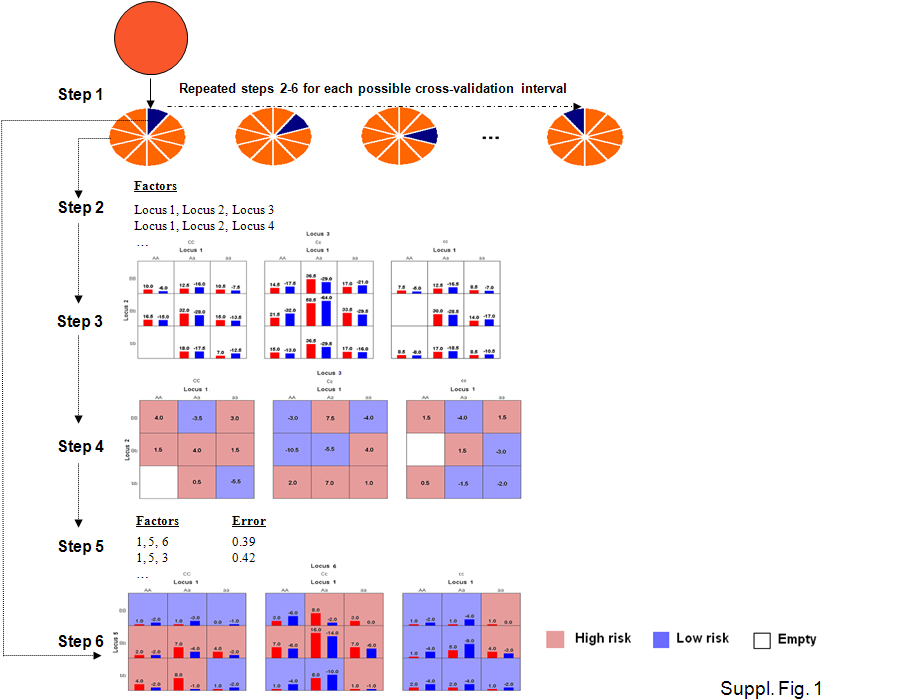

Supplement: Figure S1 — Six steps involved in data reduction algorithm. (TIF) [file pone.0016981.s001.tif]

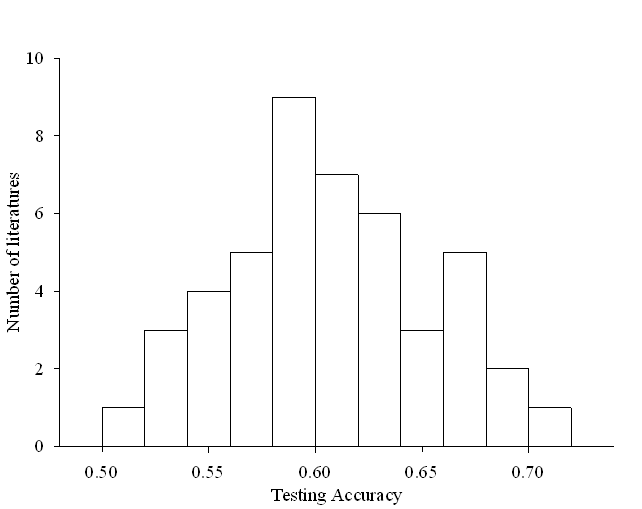

Supplement: Figure S2 — A distribution of testing accuracy from the recently reported literature on gene-gene interactions detected by the MDR or GMDR approaches, with a mean of 0.606, SD of 0.047, and range of 0.50 to 0.70 (Shapiro-Wilk test: p = 0.8033). The width of each bin is 0.02. A detailed list of these studies yielding the values used in this study is provided in Table S1. (TIF) [file pone.0016981.s002.tif]

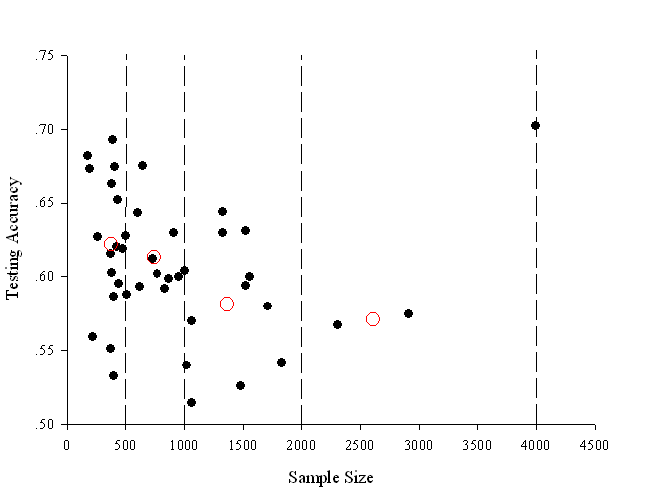

Supplement: Figure S3 — Scatter plot of reported gene-gene interactions with respect to their testing accuracy and sample sizes. The vertical lines partition the literature into four intervals with respect to their sample sizes: (0, 500), (500, 1000), (1000, 2000), and (2000, 4000). The location of each black circle is determined by the means of testing accuracy and sample size over the open spots within each interval flanked by two neighboring vertical lines. Because of the limited information available, the open circle for the sample size of≥4000 is not shown. (TIF) [file pone.0016981.s003.tif]
