# Supplementary material for: Practical and Theoretical Considerations in Study Design for Detecting Gene-Gene Interactions Using MDR and GMDR Approaches
Source: PLoS One. 2011 Feb 28;6(2):e16981. doi: 10.1371/journal.pone.0016981 (PMC3046176; doi:10.1371/journal.pone.0016981)
Supplement: Table S1 — Testing accuracy of human diseases detected with GMDR/MDR methods in the recent literature. (DOC) [file pone.0016981.s004.doc]

**Table S1: Testing accuracy of human diseases detected with GMDR/MDR methods in the recent literature**

| **Disease** | **Testing Accuracy (%)** | **No. of Loci** | **Sample Size**  **(Case/Control)** | **Literature** |
| --- | --- | --- | --- | --- |
| Alzheimer Disease | 51.43 | 2 | 506/558 | [1] |
| Asthma | 66.30 | 2 | 240/140 | [2] |
|  | 61.88 | 3 | 298/175 | [3] |
|  | 58.67 | 3 | 200/200 | [4] |
|  | 64.30 | 5 | 300/300 | [5] |
| Atrial Fibrillation | 62.74 | 3 | 250/250 | [6] |
|  | 62.74 | 3 | 250/250 | [7] |
|  | 58.80 | 1 | 255/255 | [8] |
|  | 67.28 | 1 | 97/97 | [9] |
| Autism | 57.00 | 2 | 470 families | [10] |
|  | 66.76 | 3 | 186 families | [11] |
| Bladder Cancer | 61.22 | 4 | 368/368 | [12] |
|  | 64.40 | 4 | 696/629 | [13] |
|  | 62.98 | 3 | 696/629 | [14] |
|  | 63.00 | 2 | 355/559 | [15] |
| Breast Cancer | 60.20 | 5 | 398/372 | [16] |
|  | 58.00 | 4 | 864/845 | [17] |
| Crohn’s Disease | 62.00 | 3 | 117/310 | [18] |
| Diabetic Nephropathy | 62.70 | 2 | 144/120 | [19] |
| Glioma | 63.14 | 3 | 771/752 | [20] |
|  | 59.38 | 4 | 771/752 | [21] |
| Hypertension | 52.60 | 2 | 758/726 | [22] |
|  | 55.90 | 2 | 125/95 | [23] |
|  | 68.20 | 2 | 126/51 | [24] |
| Intracranial Aneurysms | 59.88 | 3 | 402/462 | [25] |
| Lung Cancer | 60.00 | 1 | 778/781 | [26] |
|  | 67.44 | 3 | 113/299 | [27] |
|  | 54.00 | 2 | 500/517 | [28] |
| Myocardial Infarction | 54.00 | 1 |  | [29] |
| Nicotine Dependence | 60.30 | 4 | 191/191 | [30] |
|  | 59.30 | 7 | 275/348 | [31] |
|  | 55.60 | 3 |  | [32] |
| Open-angle Glaucoma | 55.14 | 6 | 176/200 | [33] |
| Oral Premalignant Lesions | 65.20 | 3 | 144/288 | [34] |
| Osteoporosis | 59.18 | 2 | 472/365 | [35] |
| Pancreatic Cancer | 57.00 | 2 | 206/860 | [36] |
| Preterm Birth | 61.58 | 2 | 172/198 | [37] |
| Prostate Cancer | 56.72 | 4 | 1444/866 | [38] |
|  | 61.00 | 7 | 195/514 | [39] |
| Rheumatoid Arthritis | 57.47 | 1 | 1393/1519 | [40] |
|  | 59.50 | 2 | 257/181 | [41] |
| Schizophrenia | 69.25 | 3 | 253/140 | [42] |
|  | 60.00 | 4 | 507/450 | [43] |
|  | 60.40 | 3 | 488/516 | [44] |
| Sporadic Breast Cancer | 53.27 | 4 | 200/200 | [45] |
| Thrombotic Stroke | 70.25 | 4 | 2000/2000 | [46] |
| Type 2 Diabetes | 54.20 | 2 | 714/1120 | [47] |
|  | 67.5 | 2 | 342/305 | [48] |
|  | 56.00 | 2 | 1162 | [49] |

**References**

1. Liang X, Slifer M, Martin ER, Schnetz-Boutaud N, Bartlett J, et al. (2009) Genomic convergence to identify candidate genes for Alzheimer disease on chromosome 10. Hum Mutat 30: 463-471.

2. Chan IH, Leung TF, Tang NL, Li CY, Sung YM, et al. (2006) Gene-gene interactions for asthma and plasma total IgE concentration in Chinese children. J Allergy Clin Immunol 117: 127-133.

3. Chan IH, Tang NL, Leung TF, Huang W, Lam YY, et al. (2008) Study of gene-gene interactions for endophenotypic quantitative traits in Chinese asthmatic children. Allergy 63: 1031-1039.

4. Millstein J, Conti DV, Gilliland FD, Gauderman WJ (2006) A testing framework for identifying susceptibility genes in the presence of epistasis. Am J Hum Genet 78: 15-27.

5. Lee JH, Moore JH, Park SW, Jang AS, Uh ST, et al. (2008) Genetic interactions model among Eotaxin gene polymorphisms in asthma. J Hum Genet 53: 867-875.

6. Tsai CT, Lai LP, Lin JL, Chiang FT, Hwang JJ, et al. (2004) Renin-angiotensin system gene polymorphisms and atrial fibrillation. Circulation 109: 1640-1646.

7. Moore JH, Gilbert JC, Tsai CT, Chiang FT, Holden T, et al. (2006) A flexible computational framework for detecting, characterizing, and interpreting statistical patterns of epistasis in genetic studies of human disease susceptibility. J Theor Biol 241: 252-261.

8. Motsinger AA, Donahue BS, Brown NJ, Roden DM, Ritchie MD (2006) Risk factor interactions and genetic effects associated with post-operative atrial fibrillation. Pac Symp Biocomput: 584-595.

9. Asselbergs FW, Moore JH, van den Berg MP, Rimm EB, de Boer RA, et al. (2006) A role for CETP TaqIB polymorphism in determining susceptibility to atrial fibrillation: a nested case control study. BMC Med Genet 7: 39.

10. Ma DQ, Whitehead PL, Menold MM, Martin ER, Ashley-Koch AE, et al. (2005) Identification of significant association and gene-gene interaction of GABA receptor subunit genes in autism. Am J Hum Genet 77: 377-388.

11. Coutinho AM, Sousa I, Martins M, Correia C, Morgadinho T, et al. (2007) Evidence for epistasis between SLC6A4 and ITGB3 in autism etiology and in the determination of platelet serotonin levels. Hum Genet 121: 243-256.

12. Zhang Z, Wang S, Wang M, Tong N, Fu G (2008) Genetic variants in RUNX3 and risk of bladder cancer: a haplotype-based analysis. Carcinogenesis 29: 1973-1978.

13. Chen M, Kamat AM, Huang M, Grossman HB, Dinney CP, et al. (2007) High-order interactions among genetic polymorphisms in nucleotide excision repair pathway genes and smoking in modulating bladder cancer risk. Carcinogenesis 28: 2160-2165.

14. Huang M, Dinney CP, Lin X, Lin J, Grossman HB, et al. (2007) High-order interactions among genetic variants in DNA base excision repair pathway genes and smoking in bladder cancer susceptibility. Cancer Epidemiol Biomarkers Prev 16: 84-91.

15. Andrew AS, Nelson HH, Kelsey KT, Moore JH, Meng AC, et al. (2006) Concordance of multiple analytical approaches demonstrates a complex relationship between DNA repair gene SNPs, smoking and bladder cancer susceptibility. Carcinogenesis 27: 1030-1037.

16. Briollais L, Wang Y, Rajendram I, Onay V, Shi E, et al. (2007) Methodological issues in detecting gene-gene interactions in breast cancer susceptibility: a population-based study in Ontario. BMC Med 5: 22.

17. Milne RL, Fagerholm R, Nevanlinna H, Benitez J (2008) The importance of replication in gene-gene interaction studies: multifactor dimensionality reduction applied to a two-stage breast cancer case-control study. Carcinogenesis 29: 1215-1218.

18. Okazaki T, Wang MH, Rawsthorne P, Sargent M, Datta LW, et al. (2008) Contributions of IBD5, IL23R, ATG16L1, and NOD2 to Crohn's disease risk in a population-based case-control study: evidence of gene-gene interactions. Inflamm Bowel Dis 14: 1528-1541.

19. Hsieh CH, Liang KH, Hung YJ, Huang LC, Pei D, et al. (2006) Analysis of epistasis for diabetic nephropathy among type 2 diabetic patients. Hum Mol Genet 15: 2701-2708.

20. Liu Y, Zhou K, Zhang H, Shugart YY, Chen L, et al. (2008) Polymorphisms of LIG4 and XRCC4 involved in the NHEJ pathway interact to modify risk of glioma. Hum Mutat 29: 381-389.

21. Liu Y, Zhang H, Zhou K, Chen L, Xu Z, et al. (2007) Tagging SNPs in non-homologous end-joining pathway genes and risk of glioma. Carcinogenesis 28: 1906-1913.

22. Kohara K, Tabara Y, Nakura J, Imai Y, Ohkubo T, et al. (2008) Identification of hypertension-susceptibility genes and pathways by a systemic multiple candidate gene approach: the millennium genome project for hypertension. Hypertens Res 31: 203-212.

23. Velez DR, Guruju M, Vinukonda G, Prater A, Kumar A, et al. (2006) Angiotensinogen promoter sequence variants in essential hypertension. Am J Hypertens 19: 1278-1285.

24. Williams SM, Ritchie MD, Phillips JA, 3rd, Dawson E, Prince M, et al. (2004) Multilocus analysis of hypertension: a hierarchical approach. Hum Hered 57: 28-38.

25. Akagawa H, Narita A, Yamada H, Tajima A, Krischek B, et al. (2007) Systematic screening of lysyl oxidase-like (LOXL) family genes demonstrates that LOXL2 is a susceptibility gene to intracranial aneurysms. Hum Genet 121: 377-387.

26. Cao G, Lu H, Feng J, Shu J, Zheng D, et al. (2008) Lung cancer risk associated with Thr495Pro polymorphism of GHR in Chinese population. Jpn J Clin Oncol 38: 308-316.

27. Chang JS, Wrensch MR, Hansen HM, Sison JD, Aldrich MC, et al. (2008) Nucleotide excision repair genes and risk of lung cancer among San Francisco Bay Area Latinos and African Americans. Int J Cancer 123: 2095-2104.

28. Li Y, Jin G, Wang H, Liu H, Qian J, et al. (2007) Polymorphisms of CAK genes and risk for lung cancer: a case-control study in Chinese population. Lung Cancer 58: 171-183.

29. Coffey CS, Hebert PR, Krumholz HM, Morgan TM, Williams SM, et al. (2004) Reporting of model validation procedures in human studies of genetic interactions. Nutrition 20: 69-73.

30. Lou XY, Chen GB, Yan L, Ma JZ, Zhu J, et al. (2007) A generalized combinatorial approach for detecting gene-by-gene and gene-by-environment interactions with application to nicotine dependence. Am J Hum Genet 80: 1125-1137.

31. Li MD, Lou XY, Chen G, Ma JZ, Elston RC (2008) Gene-gene interactions among CHRNA4, CHRNB2, BDNF, and NTRK2 in nicotine dependence. Biol Psychiatry 64: 951-957.

32. Lou XY, Chen GB, Yan L, Ma JZ, Mangold JE, et al. (2008) A combinatorial approach to detecting gene-gene and gene-environment interactions in family studies. Am J Hum Genet 83: 457-467.

33. Jia LY, Tam PO, Chiang SW, Ding N, Chen LJ, et al. (2009) Multiple gene polymorphisms analysis revealed a different profile of genetic polymorphisms of primary open-angle glaucoma in northern Chinese. Mol Vis 15: 89-98.

34. Wang Y, Spitz MR, Lee JJ, Huang M, Lippman SM, et al. (2007) Nucleotide excision repair pathway genes and oral premalignant lesions. Clin Cancer Res 13: 3753-3758.

35. Huang QY, Li GHY, Kung AWC (2009) Multiple osteoporosis susceptibility genes on chromosome 1p36 in Chinese. Bone 44: 984-988.

36. Duell EJ, Bracci PM, Moore JH, Burk RD, Kelsey KT, et al. (2008) Detecting pathway-based gene-gene and gene-environment interactions in pancreatic cancer. Cancer Epidemiol Biomarkers Prev 17: 1470-1479.

37. Velez DR, Fortunato SJ, Thorsen P, Lombardi SJ, Williams SM, et al. (2008) Preterm birth in Caucasians is associated with coagulation and inflammation pathway gene variants. PLoS ONE 3: e3283.

38. Xu J, Lowey J, Wiklund F, Sun J, Lindmark F, et al. (2005) The interaction of four genes in the inflammation pathway significantly predicts prostate cancer risk. Cancer Epidemiol Biomarkers Prev 14: 2563-2568.

39. Beuten J, Gelfond J, Franke J, Weldon K, Crandall A, et al. (2009) Single and multigenic analysis of the association between variants in 12 steroid hormone metabolism genes and risk of prostate cancer. Cancer Epidemiol Biomarkers Prev 18: 1869-1880.

40. Mei L, Li X, Yang K, Cui J, Fang B, et al. (2007) Evaluating gene x gene and gene x smoking interaction in rheumatoid arthritis using candidate genes in GAW15. BMC Proc 1 Suppl 1: S17.

41. Julia A, Moore J, Miquel L, Alegre C, Barcelo P, et al. (2007) Identification of a two-loci epistatic interaction associated with susceptibility to rheumatoid arthritis through reverse engineering and multifactor dimensionality reduction. Genomics 90: 6-13.

42. Qin S, Zhao X, Pan Y, Liu J, Feng G, et al. (2005) An association study of the N-methyl-D-aspartate receptor NR1 subunit gene (GRIN1) and NR2B subunit gene (GRIN2B) in schizophrenia with universal DNA microarray. Eur J Hum Genet 13: 807-814.

43. Shifman S, Levit A, Chen ML, Chen CH, Bronstein M, et al. (2006) A complete genetic association scan of the 22q11 deletion region and functional evidence reveal an association between DGCR2 and schizophrenia. Hum Genet 120: 160-170.

44. Zhang F, Xu Y, Liu P, Fan H, Huang X, et al. (2008) Association analyses of the interaction between the ADSS and ATM genes with schizophrenia in a Chinese population. BMC Med Genet 9: 119.

45. Ritchie MD, Hahn LW, Roodi N, Bailey LR, Dupont WD, et al. (2001) Multifactor-dimensionality reduction reveals high-order interactions among estrogen-metabolism genes in sporadic breast cancer. Am J Hum Genet 69: 138-147.

46. Shen CD, Zhang WL, Sun K, Wang YB, Zhen YS, et al. (2007) Interaction of genetic risk factors confers higher risk for thrombotic stroke in male Chinese: a multicenter case-control study. Ann Hum Genet 71: 620-629.

47. Qi L, van Dam RM, Asselbergs FW, Hu FB (2007) Gene-gene interactions between HNF4A and KCNJ11 in predicting Type 2 diabetes in women. Diabet Med 24: 1187-1191.

48. Pinelli M, Giacchetti M, Acquaviva F, Cocozza S, Donnarumma G, et al. (2006) Beta2-adrenergic receptor and UCP3 variants modulate the relationship between age and type 2 diabetes mellitus. BMC Med Genet 7: 85.

49. Pae C, Drago A, Forlani M, Patkar A, Serretti A (2010) Investigation of an epistastic effect between a set of TAAR6 and HSP-70 genes variations and major mood disorders. Am J Med Genet B Neuropsychiatr Genet 153B: 680-683.
