# Supplementary material for: Practical and Theoretical Considerations in Study Design for Detecting Gene-Gene Interactions Using MDR and GMDR Approaches
Source: PLoS One. 2011 Feb 28;6(2):e16981. doi: 10.1371/journal.pone.0016981 (PMC3046176; doi:10.1371/journal.pone.0016981)
Supplement: Text S1 — Conditional genotype distribution of the checkerboard model. (DOC) [file pone.0016981.s005.doc]

**Text S1**

**Conditional genotype distribution of the checkerboard model**

Penetrance where, , and , respectively. when {AABb, AaBB, Aabb, aaBb} for the high-risk genotypes, and for the rest genotypes when {AABB, AAbb, AaBb, aaBB, aabb}. Take MAF=0.5 and assume Hardy-Weinberg equilibrium, the frequencies of the nine genotypes can be calculated. has the form of the normal distribution , and the probability density function .

AABb and AABB are employed for high- and low-risk genotypes, respectively. Using numerical solution to integrate the expressions of the penetrances,

Consider the case sample first, by repeated application of Bayes’s theorem, for genotype ,

Similarly, for the control sample,

The rest of genotypic frequencies in the case and control samples can be calculated, respectively. After multiplying the frequencies by sample size, the distribution illustrated in Figure 2 can be calculated.
