# Supplementary material for: Practical and Theoretical Considerations in Study Design for Detecting Gene-Gene Interactions Using MDR and GMDR Approaches
Source: PLoS One. 2011 Feb 28;6(2):e16981. doi: 10.1371/journal.pone.0016981 (PMC3046176; doi:10.1371/journal.pone.0016981)
Supplement: Text S2 — The expectation of the residual score for a subject. (DOC) [file pone.0016981.s006.doc]

**Text S2**

**The expectation of the residual score for a subject**

is nonlinear as where and are MLE under the null distribution, yielding and for the case demonstrated. According to the definition of mathematical expectation, ， where , the probability a subject being affected. When is not guaranteed,

where is the normalization factor. Consider an affected subject first. The penetrance function makes every subject bearing genotype and covariate has the probability of being affected.

Take AABb and AABB as representatives for high- and low-risk genotypes, the estimated score for the subjects affected, respectively

and, for unaffected ones,, the probability a subjected being unaffected

When the expectations are calculated above, it is easy to calculate the sum of score for each genotypic cell. For example, the sum over the scores for the affected subjects in cell AABb is

and, for subjects unaffected

is estimated from genotypic distribution that , and, similarly, can apply to the rest genotypic cells, respectively.
